# Pancreatic duct occlusion after endoscopic ultrasound-guided transmural pancreatic duct drainage: a pitfall and its rescue technique

**DOI:** 10.1055/a-2334-0926

**Published:** 2024-07-01

**Authors:** Anthony Rivera Gerodias, Akihisa Ohno, Yasuhiro Komori, Yosuke Minoda, Keijiro Ueda, Tomohiko Moriyama, Nao Fujimori

**Affiliations:** 1 Department of Medicine and Bioregulatory Science, Kyushu University, Graduate School of Medical Sciences, Fukuoka, Japan; 2 37064 Institute of Digestive and Liver Diseases, Department of Medicine, Saint Lukeʼs Medical Center, Quezon City, Philippines; 3 145181 International Medical Department, Kyushu University Hospital, Fukuoka, Japan

Endoscopic ultrasound-guided pancreatic duct drainage (EUS-PDD) is a procedure to access the pancreatic duct in cases of failed endoscopic retrograde cholangiopancreatography (ERCP) 11 22 . In patients with surgically altered anatomy, transmural drainage is the preferred technique 33 . A single-pigtail plastic stent designed for EUS-PDD has been developed with favorable outcomes 44 ( Fig. 1Fig. 1 ).

**Fig. 1:**
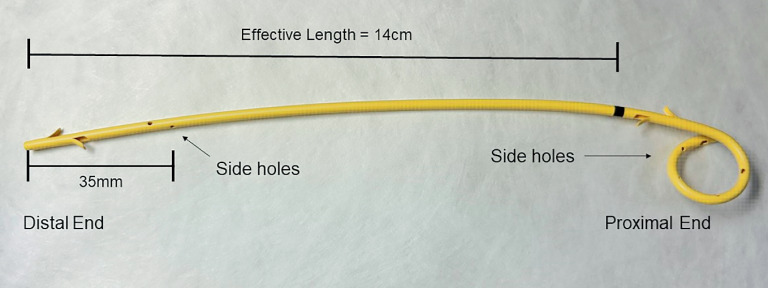
**Fig. 1** Single-pigtail plastic pancreatic duct stent designed for endoscopic ultrasound-guided pancreatic duct drainage.

A 58-year-old man with a history of pancreatic cancer who underwent pylorus-preserving pancreaticoduodenectomy 14 years previously was admitted to our institution for recurrent acute pancreatitis. Computed tomography revealed no recurrence of malignancy, pancreatic duct dilatation, or obstruction. To assess the pancreatic duct-jejunal anastomotic site, balloon ERCP was performed. The anastomotic site could not be identified, hence we performed EUS-PDD and inserted a single-pigtail stent (7 Fr, 14 cm, Type IT; Gadelius, Tokyo, Japan).

Post-procedure he developed severe abdominal pain with elevated pancreatic enzymes, leading to a diagnosis of acute pancreatitis. Evaluation presumed pancreatic duct occlusion from the stent location. A rescue procedure was performed via balloon ERCP, identifying the pancreatic duct from the distal end of the stent followed by dilatation using a balloon dilation device (4mm, REN; Kaneka, Tokyo, Japan). Finally, a single-pigtail stent was inserted (4 Fr, 5 cm; Gadelius) ( Fig. 2Fig. 2 **a–f** , Video 1Video 1 ). The procedure was successful, and he was discharged with no further recurrence of pancreatitis.

**Fig. 2:**
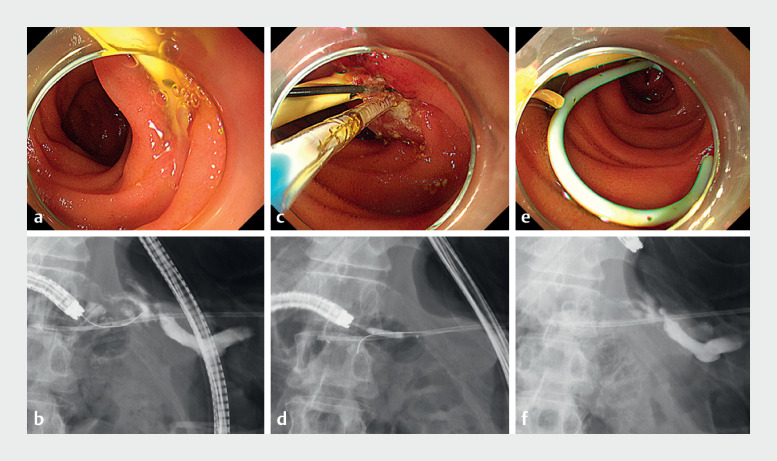
**Fig. 2a** Endoscopic view of pancreatic stent protruding from jejunal-pancreatic duct anastomotic site. **b** Fluoroscopic view of endoscope accessing the pancreatic stent from jejunal-pancreatic duct anastomotic site. **c** Endoscopic view of mechanical dilatation of the pancreatic duct. **d** Fluoroscopic view of mechanical dilatation of the pancreatic duct. **e** Endoscopic view of post-insertion of additional pancreatic stent. **f** Fluoroscopic view post-insertion of additional pancreatic stent.

Endoscopic ultrasound-guided pancreatic duct drainage following failure of balloon endoscopic retrograde cholangiopancreatography. Stent insertion was completed; however, the patient developed acute pancreatitis post-procedure and hence a rescue procedure was performed.Video 1Video 1

We determined three contributing factors that caused ductal occlusion: the pancreatic duct was not dilated, the puncture site was near the anastomotic site, and hence the stent drainage holes were located outside the pancreatic duct ( Fig. 3Fig. 3 **a–d** ).

**Fig. 3:**
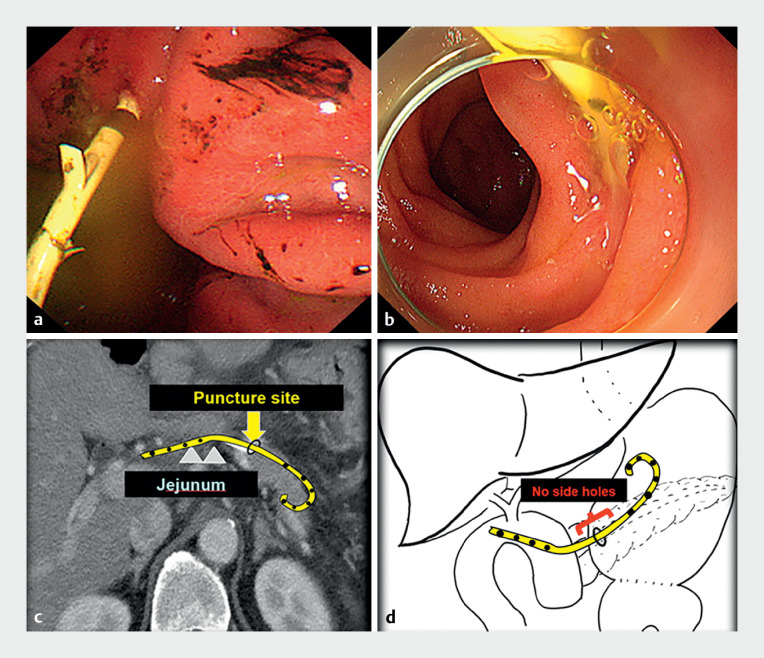
**Fig. 3a** Endoscopic view of pancreatic stent from the gastric side with side holes visible outside the pancreatic duct. **b** Endoscopic view of pancreatic stent from jejunal side with side holes visible outside the pancreatic duct. **c** Computed tomography image of puncture site with an illustration of the stent in place. **d** Illustration representing puncture site and position of pancreatic stent.

To our knowledge, this is the first report of duct occlusion occurring after EUS-PDD. In conclusion, careful consideration of the puncture site and awareness of the side hole location are key for prevention. Additionally, a rescue procedure is feasible with insertion of an additional stent for optimal drainage.

Endoscopy_UCTN_Code_CPL_1AL_2AD

## Notes

Endoscopy E-Videos https://eref.thieme.de/e-videos
*E-Videos* is an open access online section of the journal *Endoscopy* , reporting on interesting cases and new techniques in gastroenterological endoscopy. All papers include a high-quality video and are published with a Creative Commons CC-BY license. Endoscopy E-Videos qualify for HINARI discounts and waivers and eligibility is automatically checked during the submission process. We grant 100% waivers to articles whose corresponding authors are based in Group A countries and 50% waivers to those who are based in Group B countries as classified by Research4Life (see: https://www.research4life.org/access/eligibility/ ). This section has its own submission website at https://mc.manuscriptcentral.com/e-videos .
